# Relationship between High Red Cell Distribution Width and Systemic Inflammatory Response Syndrome after Extracorporeal Circulation

**DOI:** 10.21470/1678-9741-2017-0023

**Published:** 2017

**Authors:** Harsh Sateesh Seth, Prashant Mishra, Jayant V. Khandekar, Chaitanya Raut, Chandan Kumar Ray Mohapatra, Ganesh Kumar K. Ammannaya, Jaskaran Singh Saini, Vaibhav Shah

**Affiliations:** 1 Department of Cardiovascular and Thoracic Surgery Lokmanya Tilak Municipal Medical College and General Hospital

**Keywords:** Erythrocyte Indices, Extracorporeal Circulation, Systemic Inflammatory Response Syndrome

## Abstract

**Objective:**

Cardiac surgical operations involving extracorporeal circulation may develop severe inflammatory response. This severe inflammatory response syndrome (SIRS) is usually associated with poor outcome with no predictive marker. Red cell distribution width (RDW) is a routine hematological marker with a role in inflammation. We aim to determine the relationship between RDW and SIRS through our study.

**Methods:**

A total of 1250 patients who underwent cardiac surgery with extracorporeal circulation were retrospectively analyzed out of which 26 fell into the SIRS criteria and 26 consecutive control patients were taken. RDW, preoperative clinical data, operative time and postoperative data were compared between SIRS and control groups.

**Results:**

The demographic profile of the patients was similar. RDW was significantly higher in the SIRS *versus* control group (15.5±2.0 *vs.* 13.03±1.90), respectively with *P* value <0.0001. There was significant mortality in the SIRS group, 20 (76.92%) as compared to 2 (7.6%) in control group with a *P* value of <0.005. Multiple logistic regression analysis revealed that there was significant association with high RDW and development of SIRS after extracorporeal circulation (OR for RDW levels exceeding 13.5%; 95% CI 1.0-1.2; *P*<0.05).

**Conclusion:**

Increased RDW was significantly associated with increased risk of SIRS after extracorporeal circulation. Thus, RDW can act as a useful tool to predict SIRS in patients undergoing cardiac surgery with extracorporeal circulation. Hence, more aggressive measures can be taken in patients with high RDW to prevent postoperative morbidity and mortality.

**Table:** 

Abbreviations, acronyms & symbols				
ACT	= Activated clotting time		IL-6	= Interleukin-6
AMI	= Acute myocardial infarction		MCHC	= Mean cell hemoglobin concentration
CABG	= Coronary artery bypass grafting		MCV	= Mean corpuscular volume
CBC	= Complete blood count		MOD	= Multi Organ Dysfunction
CPB	= Cardiopulmonary bypass		PaCO_2_	= Partial arterial carbon dioxide pressure
ECC	= Extracorporeal circulation		RBC	= Red blood cell
ET	= Endothelin		RDW	= Red cell distribution width
ESR	= Erythrocyte sedimentation rate		ROC	= Receiver operator curve
EuroSCORE	= European system for cardiac operative risk evaluation		SD	= Standard deviation
HCT	= Hematocrit		SIRS	= Severe inflammatory response syndrome
hs-CRP	= High-sensitive C-reactive protein		TNF-alpha	= Tumor necrosis factor-alpha
ICU	= Intensive care unit		WBC	= White blood cell

## INTRODUCTION

Cardiac surgery using cardiopulmonary bypass (CPB) provokes a systemic inflammatory response. This is mainly triggered by contact activation of blood by artificial surfaces of the extracorporeal circuit. Although often remaining subclinical and resolving promptly at the end of CPB, in its most extreme form this inflammatory response may be associated with the development of the systemic inflammatory response syndrome (SIRS) that can often lead to multi organ dysfunction (MOD) and death^[1]^.

Red cell distribution width (RDW) is a quantitative measure of anisocytosis, the variability in size of the circulating erythrocytes. It is routinely measured by automated haematology analysers and is reported as a component of the complete blood count (CBC)^[2]^.

RDW is a recently described novel biomarker that has been shown to be predictive of adverse outcomes in multiple cardiovascular disease settings, including stable coronary artery disease, chronic heart failure and acute myocardial infarction (AMI)^[3-5]^. Although the plausible pathobiological mechanisms explaining the relationship of RDW with adverse cardiovascular outcomes are yet to be elucidated, both inflammation and oxidative stress are believed to play a role^[6]^.

The molecular basis of the above mentioned association has been mainly attributed to the ability of RDW's capability to reliably reflect an increase in the levels of circulating cytokines, such as Interleukin-6 (IL-6), Tumor Necrosis Factor-alpha (TNF-alpha) and hepcidin^[7]^.

SIRS is a dreaded complication of any surgery. It is known to have a poor prognosis and in the current scenario there are no useful laboratory and clinical parameters to predict it. There has not been much work done elucidating the possible association between high RDW and development of SIRS after extracorporeal circulation (ECC).

## METHODS

After approval of the study by ethics committee of our institution (IEC/67/16), we retrospectively evaluated 1250 patients who underwent elective cardiac surgery with ECC from August 2012 to August 2016. Two groups were formed (SIRS and control groups), according to the following criteria.

### SIRS Group

According to this criterion, we identified 26 patients with SIRS, which presented with two or more of the following features:

- Temperature ≥38ºC or ≤36ºC; Heart rate >90 beats/min.
- Respiratory rate >20 breaths/min or partial arterial carbon dioxide pressure (PaCO_2_) <32 mmHg.
- White blood cell (WBC) count ≥12,000/µl or ≤ 4,000/µl^[8]^.

Exclusion criteria: Mechanical ventilation more than 48 hours before surgery, preoperative infection, death during surgical intervention or in the first 48 hours after surgery, proved postoperative infection within the first 5 days, record with incomplete data.

### Control Group

Twenty-six consecutive patients with similar demographic parameters who underwent cardiac surgery with ECC were included in the study and who met the inclusion criteria (elective operation, no preoperative infection, no coagulopathy, ejection fraction >35%). These patients were operated in the similar timeframe as that of the corresponding SIRS patient. Patients who developed SIRS were excluded from the control group.

### Data Collection

Demographic parameters were recorded as the patients were included in the study: age, gender, weight, left ventricular ejection fraction, European system for cardiac operative risk evaluation (EuroSCORE), hematological and biochemical parameters. Perioperative data were taken as: type of surgery, cross-clamp time, ECC duration and oxygenator type. Postoperative collected data were also taken into consideration and recorded as: need for inotropic support, postoperative complications such as SIRS, acute kidney injury (increased serum creatininemia ≥1.5 or urine output <0.6 ml/kg/h during six consecutive hours)^[9]^ and mortality. Clinical signs such as heart rate, body temperature and respiration rate were also recorded hourly in the intensive care unit (ICU). Serial arterial blood gas analysis was done in the ICU.

### Hematologic and Biochemical Measurements

The complete blood count (CBC) and biochemistry panel of our patients were measured routinely after 12 hours from fasting at the time of admission. Baseline RDW values were measured with the use of the Sysmex XT-2000i Automated Hematology Analyzer (Roche Diagnostics, Mannheim, Germany) in our hospital's laboratory and were reported as a coefficient of variation (percentage) of red blood cell (RBC) volume. The normal reference range for RDW in our laboratory is between 11.6% and 14.8%.

### Operative details

The same anesthesiology and surgical team operated all the patients. Standard median sternotomy incision with aortic cannulation, single or bicaval cannulation with membrane oxygenator (Terumo CAPIOX) with application of single cross-clamp and administration antegrade root cold blood cardioplegia with mild to moderate systemic hypothermia (28-32ºC) in all patients. All patients were heparinized with 300 units/kg before establishment of ECC. Activated clotting time (ACT) was checked at 15-minute intervals until the target ACT of 450-600 seconds was achieved which is when CPB was initiated. All patients who underwent coronary artery bypass grafting (CABG) had at least one arterial graft (left internal mammary artery) as well as venous grafts (great saphenous vein). Proximal anastomosis was done on a beating heart using a side-biting clamp. Valve replacements were performed using pledgeted sutures in horizontal mattress fashion. All patients received antibiotic prophylaxis at the time of induction with cefazolin sodium 30 mg/kg at induction and repeated every four hours during surgery.

### Statistical Analysis

Statistical analysis was performed using MedCalc statistical software version 10.3.0.0 for Windows. Data are presented as the mean ± standard deviation (SD) for continuous variables and percentage for categorical variables. Student's t-test was used to compare continuous variables and the chi-square test was used for categorical variables. A *P*-value <0.05 indicates statistical significance. Multiple logistic regression analysis was used to evaluate the independent predictors of SIRS occurrence.

## RESULTS

After the retrospective analysis of patients as per the inclusion and exclusion criteria, 26 patients came under SIRS group and 26 consecutive patients were assigned in the control group.

The distribution of inclusion criteria was as follows: temperature (>38ºC) in 5 (19.2%) patients, heart rate >90/min in 18 (69.2%) patients, respiratory rate abnormalities in 22 (84.6%) patients and WBC criteria in 10 (38.4%) patients.

Baseline clinical and demographic parameters are summarized in Table 1.

**Table 1:** Baseline clinical and demographic parameters.

Parameter	SIRS group (n=26)	Control group (n=26)	*P* value
Age	61.66±9.2	56.8±19.2	0.250
Gender (F/M)	10 -16	12 -14	0.574
Diabetes mellitus	8	3	0.085
Hypercholesterolemia	5	3	0.447
Ejection fraction	48.5±9.5	50.5±9.5	0.566
Active smoker	11	10	0.777
Hypertension	8	7	0.759
Chronic obstructive lung disease	9	6	0.358
EuroSCORE	4.52±2.38	4.10±2.20	0.503

SIRS=systemic inflammatory response syndrome; F=female; M=male

**Fig. 1:**
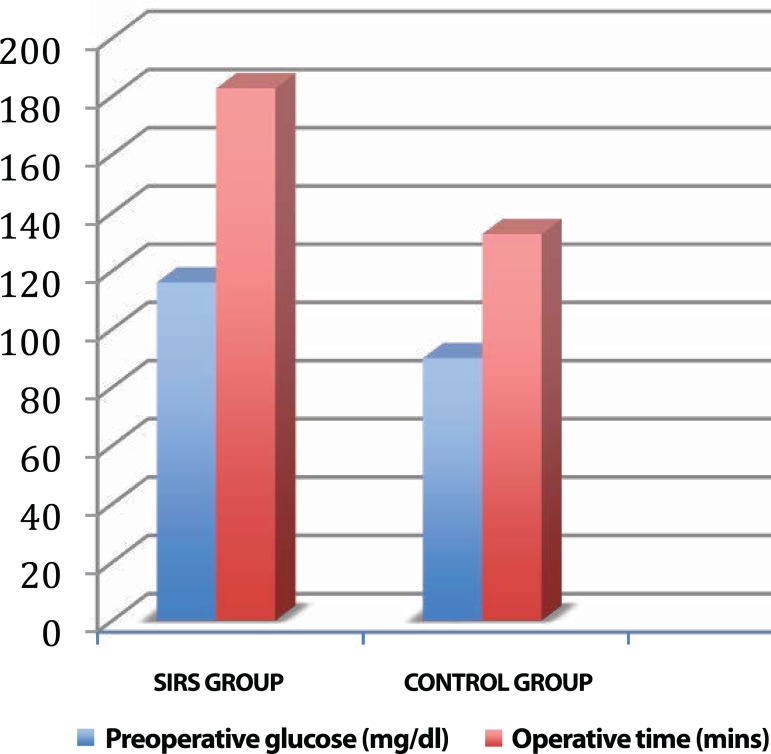
Graphical representation of preoperative glucose levels and operation time between SIRS and control groups.

**Fig. 2:**
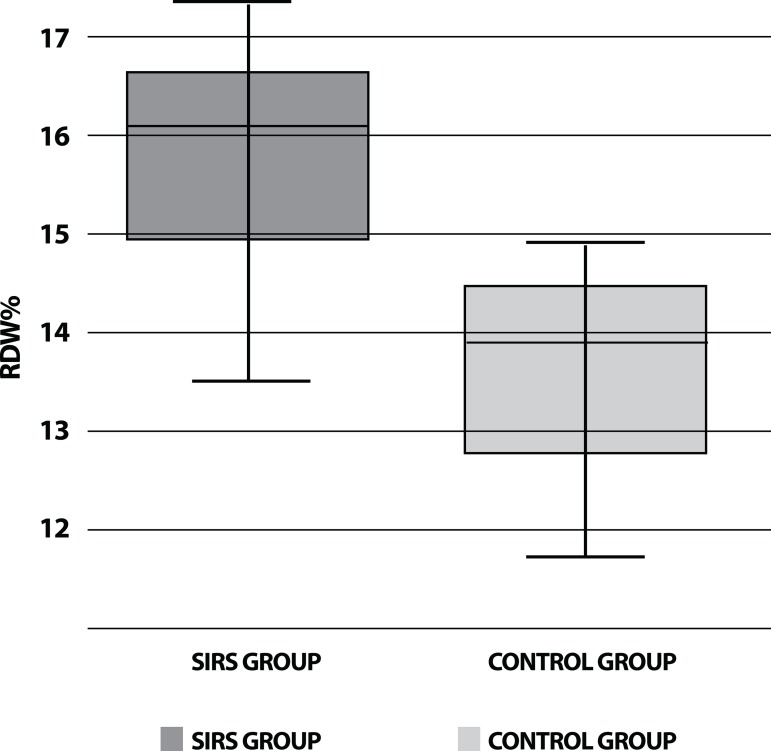
Comparison of levels of red cell distribution width in SIRS and control groups.

Both groups were homogeneous in terms of age, gender, smoking habits, presence of diabetes mellitus and predisposition to allergy, presence of hypercholesterolemia, preoperative ejection fraction and the presence of chronic obstructive lung disease, hypertension and EuroSCORE. Operative data of the patients are summarized in Table 2. We found significant differences between the two groups regarding total operation time, number of units of blood transfused and need for inotropic support. Significant postoperative mortality occurred among the SIRS group patients of whom 20 (76.92%) were lost, whereas this turned out to be 2 (7.6%) in the control group (*P*<0.001). The hematological parameters are presented in Table 3. WBC, RBC, hematocrit (HCT), mean corpuscular volume (MCV), mean cell hemoglobin concentration (MCHC), platelet, and creatinine levels were similar in both groups. Of the total 1250 patients, 33 of them had high preoperative RDW. Out of the 26 patients who developed SIRS, 22 of them had high preoperative RDW values. However, RDW was significantly higher in the SIRS group *versus* the control group (15.5±2.0 *vs.* 13.03±1.90), respectively, *P*<0.0001. In addition, preoperative blood glucose levels were also found to be significantly higher in the SIRS group *versus* the control group (116±22 *vs.* 90±14), respectively, *P*<0.0001 (Figure 1). Multiple logistic regression analyses showed an association between high RDW levels and SIRS development (OR for RDW levels exceeding 13.5%; 95% CI 1-1.2; *P*<0.05) (Figure 2). The receiver operator curve (ROC) analysis suggested that the optimum cut-off level of RDW for SIRS was 12.9% (sensitivity: 93.74%; specificity: 76%; area under the curve: 0.851, *P*<0.05).

**Table 2:** Operative data of both groups.

Parameter	SIRS group (n=26)	Control group (n=26)	*P*value
Combined surgery (coronary/valve)	8	6	0.531
Isolated valve	8	10	0.559
Isolated CABG	10	10	1.000
Operation time (min)	182.7±44.2	132.6±38.8	0.001*
Cardiopulmonary bypass time (min)	136±40.3	121±30.5	0.136
Cross-clamp time (min)	98.3±40.6	88.2±20.8	0.264
Need for inotropic support	23	8	0.0002*
Number of blood transfusion (unit)	2±1.2	1.2±0.8	0.006*
Intra-aortic balloon pump	3	1	0.297
Major complications	__	__	0

CABG=coronary artery bypass grafting; SIRS=systemic inflammatory response syndrome

* Statistically significant

**Table 3:** Laboratory parameters.

Parameter	SIRS group (n=26)	Control group (n=26)	*P*value
WBC (×10^3^/mm^3^) (NR 4-11)	9.42±3.75	8.34±3.55	0.291
RBC (×10^6^/mm^3^) (NR 4.2-5.7)	4.64±0.754	4.44±0.88	0.382
Platelet (×10^3^/mm^3^) (NR 150-400)	245±85.66	242±83.33	0.898
HCT (%) (NR 35-50)	38±5.8	37±5.4	0.522
MCV (fL) (NR 77-96)	84.44±6.07	86.55±7.06	0.253
MCHC g Hb/dl (32-36)	33±1.5	33±1.7	1.00
RDW (%) (NR 11.6-14.8)	15.5±2	13.03±1.90	0.0001*
PO blood creatinine (mg/dl) (NR 0.5-1.5)	0.98±0.23	0.97±0.32	0.897
PO fasting blood glucose (mg/dl) (NR 76-110)	116±22	90±14	0.0001*

Hb=hemoglobin; HCT=hematocrit; NR=normal range; RBC=red blood cell; RDW=red cell distribution width; SIRS=systemic inflammatory response syndrome; WBC=white blood cell; MCV=mean corpuscular volume; MCHC=mean cell hemoglobin concentration; PO=postoperative.

* Statistically significant

## DISCUSSION

This study shows us the importance of preoperative RDW as a predictive marker for development of SIRS after ECC. RDW is a routine parameter available in complete blood count.

This syndrome occurs in about 0.5-1.7% of patients after ECC and can be associated with multiple organ failure which has a mortality of 40-60%, which can also be higher^[10]^. Although perioperative SIRS occurs in about 2% of all ECC procedures, the mortality is high and comparable to that of severe sepsis^[11]^.

In our study, the incidence of SIRS was 2.08% of the patients with a mortality of 76.92%. The patients who expired in the SIRS group ultimately had multiorgan failure. ECC is crucial part of many cardiac surgical operations. Multiple factors associated with the use of CPB contribute toward the generation of perioperative SIRS. These include the generation of shear forces from roller pumps driving blood through the bypass circuit, hypothermia as blood is passed through the extracorporeal circuit, and contact activation of plasma protein systems as circulating blood is exposed to artificial surfaces in the bypass circuit. The generation and release of endogenous inflammatory mediators leading to the development of SIRS follow this^[1]^. The main underlying molecular mechanisms of such inflammation are activation of the complement system, increasing production of cytokines, oxygen radicals, release of endothelin (ET) and the expression of adhesion molecules on leukocytes and the endothelium^[12,13]^.

In 2007, Felker et al.^[13]^ firstly discovered that increasing RDW is an independent predictor for the prognosis of heart failure patients, and researchers gradually discovered that RDW is closely associated with the prognosis of cardiovascular diseases. Recent studies showed that increasing RDW is not only a predictor for poor prognosis of heart failure. But it also exhibits a predictive value towards the prognosis of stable coronary artery disease patients who have underwent percutaneous coronary intervention therapy.

Red cell differentiation is also related to oxidative stress and to the release of cytokines in response to inflammation induced by cardiac surgery^[14]^. Oxidative stress directly damages erythrocytes and leads to shortened erythrocyte survival, resulting in elevated RDW^[15]^. Lippi et al.^[16]^ showed a correlation between RDW and indices of inflammation, such as elevated erythrocyte sedimentation rate (ESR) and high-sensitive C-reactive protein (hs-CRP), identifying a strong and graded increase in both ESR and hs-CRP across various RDW values. In a study by Semba et al.^[17]^ it was found that antioxidant status might influence RDW and play a role in the relationship between increased RDW and worsened clinical prognosis. These cytokines attenuate the activity of erythropoietin and cause the production of ineffective red blood cells, leading to elevated RDW^[18]^.

Perlstein et al.^[19]^ showed that RDW strongly predicted all-cause and cardiovascular mortality. Lappé et al.^[20]^ demonstrated that RDW was associated with mortality in patients with stable coronary disease and in normal coronary subjects. In addition, RDW is also an independent prognostic factor for patients with peripheral arterial disease. In one study, a 10% increased risk of mortality was observed with a 1% increase in RDW^[21]^. In our study, patients with a high RDW value over 13.5% had increased incidence of SIRS and the relation became even stronger if the RDW value was more than 15%. In a study by Kumar et al.^[22]^, patients with higher RDW had a longer ICU stay (155.6±71.3 *vs.* 122.4±61.3 hours, *P*=0.02). It was consisent with our study showing significant morbidity in the SIRS group as compared to control group in terms of length of ICU stay (158.2±72.3 hours *vs.* 100.2±44.2 hours, *P*<0.001). Cemin et al.^[23]^ also found that RDW was a significant predictor of AMI, exhibiting an area under the curve of 0.61 (95% CI, 0.54-0.68).

The sensitivity and specificity of RDW at the 13.7% cut-off value were 0.75 and 0.52, respectively. In our study, ROC analysis suggested that the optimum cut-off level of RDW for SIRS was 12.9% (sensitivity: 93.74%; specificity: 76%; area under the curve: 0.851, *P*<0.05), and the mean operation period was significantly longer in the SIRS group than the control group.

In accordance with total operation time, ECC time was found longer in the SIRS group, but it did not reach to statistical significance. Kirklin et al.^[24]^ emphasized that an increase in ECC time from 60 to 120 minutes would also increase postoperative morbidity in all age groups. There was a difference in the operating times, but the clamp time and ECC times were similar in the two groups. There was not one particular cause for the same but some of them are as follows: there was increased time taken to harvest the left internal mammary artery in few patients of CABG. In some patients, there were cardiotomy-bleeding points, which needed reinforcement sutures. In some there was bleeding from the aortic line, which needed reinforcement sutures. In some patients after coming off CPB, there was an oozy field, which needed to be addressed hence took time before chest closure. The fact that clamp time and ECC times were similar in our study suggests that the ECC time could not be considered as a confounding factor. The operating time, which was higher in the SIRS group, was the time either before onset or after termination of ECC.

**Fig. 3:**
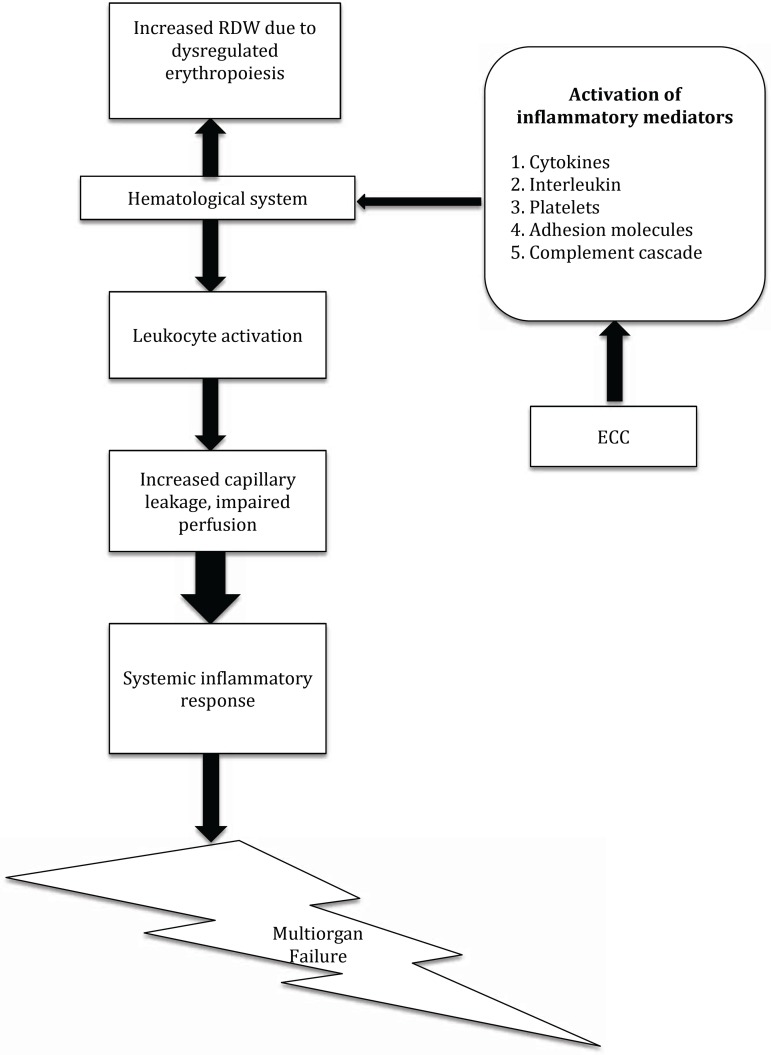
Proposed hypothesis for correlation between high red cell distribution width and SIRS.

Preoperative high blood glucose levels were also found to be significant in the SIRS group in comparison with the control group. This condition can hypothetically be explained with cardiac and pulmonary stress induced by catecholamine release, which results in increased preoperative glucose levels^[25]^. In our study, there was a significant correlation between number of units of blood transfused and development of SIRS. These results suggest that high preoperative RDW can be used as an effective predictive marker for SIRS in patients undergoing cardiac surgery with ECC. As postulated, patients with high RDW have dysregulated erythropoiesis. These patients may also have qualitative defects in their platelets, which may lead to increase bleeding after ECC. This may be the reason that our patients with high RDW required more transfusion. However, our study was not designed to establish objective evidence of qualitative platelet dysfunction and to determine the possible causes of SIRS, but we can only speculate on the possible causes (Figure 3).

The reason for high RDW is that under pulmonary or cardiovascular stress such as hypoxia or low cardiac output, there is increased cytokine level, which attenuates the activity of erythropoietin. This results in production of ineffective red blood cells leading to an elevated RDW^[23]^.

This study is retrospective and has its own limitations. The sample size is small as the occurrence of SIRS is infrequent. This study does not take into account congenital heart conditions. This is a single centre study for SIRS patients hence they are a potential hindrance to its external validity. We also realize that there were confounding factors like increased blood transfusion and increased operative time in a few patients of the SIRS group. Nevertheless, high RDW had a significant association in the development of SIRS after ECC in our study. Therefore, we also recommend that a similar study with a higher sample size, prospective design and randomized control should be done to validate these findings even further. This was not possible in our setup.

## CONCLUSION

In conclusion, the main finding to be noted is that there is a significant association between elevated RDW and development of SIRS after ECC. This finding can provide us with valuable information for predicting SIRS in patients undergoing open-heart surgery without any additional costs, as RDW is a part of routine complete blood count. This valuable piece of information can also be made to use such that we find various alternatives to achieve the best result for our patient:

(1) avoiding CPB altogether (off pump surgery);
(2) removing activated neutrophils (leukodepletion filters);
(3) using hemofiltration in appropriate patients;
(4) we as clinicians should be watchful in patients with elevated RDW and take appropriate aggressive measures.

**Table:** 

Authors' roles & responsibilities	
HSS	Substantial contributions to the conception or design of the work; or the acquisition, analysis, or interpretation of data for the work; final approval of the version to be published
PM	Substantial contributions to the conception or design of the work; or the acquisition, analysis; final approval of the version to be published
JVK	Substantial contributions to the conception or design of the work; or the acquisition; final approval of the version to be published
CR	Substantial contributions to the conception or design of the work; or the acquisition; final approval of the version to be published
CKRM	Substantial contributions to the analysis; final approval of the version to be published
GKKA	Interpretation of data for the work; final approval of the version to be published
JSS	Final approval of the version to be published
VS	Final approval of the version to be published
